# Expression analysis of cytokines IL-5, IL-6, IL-8, IL-17 and VEGF in breast cancer patients

**DOI:** 10.3389/fonc.2022.1019247

**Published:** 2022-12-01

**Authors:** Gang Liu, Xiang-Ting Chen, Hao Zhang, Xin Chen

**Affiliations:** ^1^ Department of Endocrine and Breast Surgery, The First Affiliated Hospital of Chongqing Medical University, Chongqing, China; ^2^ Department of Neurology, Bishan Hospital of Chongqing Medical University, Chongqing, China

**Keywords:** cytokines, vascular endothelial growth factor (VEGF), clinicopathological features, diagnostic efficacy, breast cancer

## Abstract

**Objective:**

To investigate the relationship between changes in peripheral blood vascular endothelial growth factor (VEGF), interleukin-5 (IL-5), interleukin-6 (IL-6), interleukin-8 (IL-8) and interleukin-17 (IL-17) concentrations in breast cancer patients and their significance and clinical value in breast cancer staging and invasive metastasis.

**Methods:**

From September 2021 to April 2022, 60 breast cancer patients from Chongqing Medical University Hospital No. 2022 were enrolled in the breast breast cancer surgery group, while 30 patients with benign breast disease were enrolled in the control group during the same period. Venous blood samples were collected at admission and 1 week after surgery to determine the expression of these factors in serum. Statistical methods such as Wilcoxon test and Spearman correlation analysis were used to analyze the relationship between the above factors and the clinicopathological characteristics of the patients.

**Results:**

By analyzing data from patients with benign and malignant breast tumors, an association was found with serum levels of IL-6, IL-17 and VEGF. Their respective areas under the operating characteristic curve were 0.649, 0.734 and 0.656 (P < 0.05). There were significant differences in the cytokine expression levels of IL-17 and VEGF in different molecular typing (P values were 0.008 and 0.040, respectively). The expression levels of IL-17 and VEGF were higher in HER-2 receptor-positive and triple-negative patients than in hormone receptor-positive patients (P < 0.05). Also, no significant correlation was found between the various cytokines mentioned in the article and breast cancer lymph node metastasis and Tumor Node Metastasis stage (TNM stage). In addition, in the breast cancer surgery group, postoperative VEGF levels were lower (P < 0.05) and IL-6 levels were higher (P < 0.05) compared to preoperative levels.

**Conclusions:**

Serum IL-6, IL-17, and VEGF are strongly associated with breast cancer development and can be used as a reference indicators for breast cancer diagnosis. In addition, post-operative VEGF levels decreases and IL-6 levels increases compared to pre-operative levels, which can also be used as an a postoperative follow-up indicator. In contrast, IL-5 and IL-8 have not found to be significantly associated with breast cancer patients in this study, which requires further study.

## Introduction

Breast cancer is the most common cancer and the second leading cause of cancer-related death among women in developed countries, with approximately 400,000 women dying from the disease each year worldwide (1), posing a serious threat to women’s lives and health, and in recent years has shown a trend toward a higher incidence in younger, non-obese populations, which is of increasing concern (2). The factors inducing breast cancer include family history of breast cancer, radiation exposure, fewer births, infertility, serum cytokine expression level, etc. Among them, human serum cytokines have a variety of biological functions. For example, it was mentioned in the literature that angiogenesis is inevitable during the growth of breast tumors and the formation of local and systemic metastasis, the high expression of VEGF is associated with poor prognosis in most cases (3). At the same time, in patients with lymph node positive and human epidermal growth factor receptor 2 positive diseases, the expression level of IL-5 usually increases and is associated with poor prognosis. The increased expression of IL-6 and IL-8 in patients without progesterone receptor expression in tumor G3 usually indicates a poor prognosis, and there is a correlation between IL-8 and neovascularization, which may promote metastatic spread (4, 5). Simultaneously, studies have shown that IL-17 is overexpressed in tumor interstitial cells of TNBC-NST, the overexpression of IL-17 may participate in the active tumor angiogenesis through its signal transduction pathway, leading to the increase of the secretion of VEGF-A in the tumor, thus promoting the tumor progression (6). The above studies suggest that the level of cytokine expression seems to be related to the differentiation and proliferation, apoptosis, metastasis and differentiation of breast cancer tumor cells. Dynamic detection of these changes of cytokine expression can reflect part of the biological characteristics of tumors at this stage.

At present, the common clinical diagnostic methods for breast cancer include X-ray mammography, breast ultrasound, breast MRI, needle aspiration cytology, puncture biopsy, etc. Imaging diagnosis is non-invasive and cannot be used as a common means of disease monitoring. Needle aspiration cytology and needle biopsy are invasive examinations, but they have limitations in the early diagnosis and monitoring of tumors. In this paper, the value of the above factors in evaluating the progress, invasion and metastasis of breast cancer was studied by measuring the level of serum cytokines, combined with disease stage and lymph metastasis, which provides an important basis for early diagnosis and dynamic monitoring of breast cancer.

## Information and methods

### Patients and data collection

We selected 66 breast cancer patients admitted to the First Hospital of Chongqing Medical University from September 2021 to May 2022 as the study population, and another 30 patients with benign breast disease attending the same period were selected as the control group for retrospective analysis. All surgical treatments were performed by surgeons with extensive experience in our center, and postoperative pathological findings were determined by two experienced pathologists in our center. Inclusion criteria (1): Histologically confirmed invasive breast cancer; (2) No history of radiotherapy, chemotherapy and biologic targeted therapy before enrollment; (3) Serum cytokine levels were measured within 1 week before surgery and 2 days after surgery, respectively. Exclusion criteria: (1) patients who had taken immunomodulators and hormonal drugs within 6 months; (2) exclusion of other major concurrent diseases, concurrent other cancers; (3) incomplete clinicopathological data. Pathological diagnosis was the gold standard for determining the status of breast cancer and anterior lymph nodes. We finally included a total of 60 breast cancer patients, all of whom received standardized surgical treatment for breast cancer, for the breast cancer group, with a mean age of (51.6 ± 9.3) years; 29 (48.3%) of them were ≥50 years old; among them, 27 were in TNM stage I, 26 in stage II, 6 in stage III, and 1 in stage IV, which were divided into 27 (45%) in the stage I group and 33 (55%) in the stage II+III+IV group. 16 (26.6%) in the group with lymph node metastasis and 44 (73.3%) in the group without lymph node metastasis according to lymph node metastasis; 34 (56.6%) in the postmenopausal group and 26 (43.3%) in the premenopausal group according to menopausal status. Among these patients, 28 (46.6%) patients were pathologically diagnosed as hormone receptor positive, 25 (41.6%) as Her-2(+), and 7 (11.6%) as triple negative.

We collected clinical and histopathological data of the patients, which included age, height, weight, BMI, menstrual status, tumor size, TNM stage, presence of lymph node metastasis, human epidermal growth factor receptor (HER-2), proliferation index Ki-67, molecular typing, pre- and post-operative levels of IL-5, IL-6, IL-8, IL-17 and VEGF. The clinicopathological characteristics of 60 patients showed (**Table 1**) that the differences in age, height, weight, Body Mass Index (BMI) and menstrual status between the two groups were not statistically significant (P > 0.05) and were comparable.

**Table 1:** baseline information table and univariate analysis.

	Total (n=90)	Benign tumor group (n=30)	Malignant tumor group (n=60)	P-value
Patients (n [%])	90[100]	30[100]	60[100]	–
Age (years)	50.40 ± 9.50	47.93 ± 9.61	51.63 ± 9.28	0.081
Height (cm)	158.00 (155.00-160.00)	158.00 (155.00-161.00)	157.50 (154.25-160.00)	0.168
Body weight (kg)	58.53 ± 7.51	58.48 ± 7.44	58.56 ± 7.60	0.965
BMI	23.66 ± 3.00	23.45 ± 3.31	23.76 ± 2.87	0.643
Menopausal status (n [%])	90 [100]	30 [100]	60 [100]	0.075
IL-5 (pg/ml)	1.01 (0.50-2.13)	1.00 (0.51-2.24)	1.01 (0.38-2.19)	0.857
IL-6 (pg/ml)	0.74 (0.39-1.98)	0.55 (0.38-1.00)	1.11 (0.45-2.44)	**0.021**
IL-8 (pg/ml)	1.43 (0.39-4.72)	1.03 (0.27-4.69)	1.47 (0.79-4.73)	0.357
IL-17 (pg/ml)	0.47 (0.20-1.08)	0.68 (0.26-2.06)	0.40 (0.18-0.88)	**0.016**
VEGF (pg/ml)	27.12 (15.24-47.96)	16.64 (9.04 - 27.97)	35.78 (20.73-75.80)	**<0.001**

Values in bold represent statistically significant results (P<0.05).

### Methods

#### Specimen collection

In 60 patients with breast cancer, 3ml of peripheral venous blood was drawn from the patients on an empty stomach in the morning within one week before operation and 48 hours after operation and placed in a disposable blood collection vessel. The samples were sent for examination immediately after being isolated from the body. Fibrinogen was fully coagulated by placing them in a 37°C water bath box for 20 to 30 minutes, and then centrifuged at 4000 rpm for 15 minutes. The serum was aspirated and tested immediately. The serum samples that could not be detected in time were frozen in - 20°C refrigerator for standby. The test indexes were cytokines (IL-5, IL-6, IL-8, IL-17) and vascular endothelial growth factor (VEGF).

#### Detection of serum cytokine levels

Cytokines (IL-5, IL-6, IL-8, IL-17) were determined using the IMMULITE 1000 chemiluminescence analyzer from Siemens, which applies the 14 cytokine assay kit, and the assay method took the flow fluorescence luminescence method. Vascular endothelial growth factor was determined by SMART 3000S fully automated chemiluminescence immunoassay analyzer from Cosmax, which applied the vascular endothelial growth factor assay kit, and the assay method took the chemiluminescence method, and the above operations were performed according to the kit instructions.Vascular endothelial growth factor assay kit was purchased from Shandong Kanghua Biomedical Technology Co.

### Statistical analysis

Statistical analyses were performed using SPSS version 26.0. Patient characteristics were expressed as frequencies or descriptive analyses. If the quantitative data conformed to a normal distribution, they were expressed as mean ± standard deviation, the statistical method of t-test for independent samples was used for comparison between groups, the paired t-test was used for comparison between before and after data. And if the quantitative data did not conform to a normal distribution, they were expressed as M (P25, P75), the statistical method of Wilcoxon (Mann-Whitney U) test was used for comparison between groups, the Kruskal-Wallis (K-W test) test was used for comparison between k-groups, and the Wilcoxon signed rank sum test was used for comparison between before and after data. Qualitative data were expressed as n (%) and the statistical method was the chi-square test. Multifactorial analysis was performed using unconditional logistic multiple regression analysis and subject operating characteristic curves (ROC curves) were used to analyse the value of the above indicators in the diagnosis of breast cancer. The area under the curve (AUC) was used as a measure to predict diagnostic effectiveness and the maximum index was used as the cut-off value. Differences were statistically significant at p<0.05.

## Results

### Single-factor analysis revealed possible causative factors for breast malignancy

The differences were no statistically significant (P≥0.05) when comparing the age, height, weight, BMI, menstrual status, IL-5 and IL-8 levels of the two groups of patients whose gender was female; the differences were statistically significant (P < 0.05) when comparing the IL-6, IL-17 and VEGF levels of the two groups of patients (**Table 1**).

### Multifactorial analysis revealed independent risk factors for breast malignancy

Using the indicators with statistically significant differences in the univariate analysis as independent variables, the results of the multifactorial logistic regression analysis showed that IL-6, IL-17 and VEGF were independent risk factors for breast malignancy (P = 0.033, 0.035, 0.021), as shown in **Table 2**.

**Table 2:** Comparison of independent risk factors for breast malignancy.

Indicators	B	SR	Wald	P-value	OR	OR 95% CI	
						Lower limit	Upper limit
IL-6	0.57	0.268	4.532	0.033	1.768	1.046	2.988
IL17	-0.136	0.064	4.467	0.035	0.873	0.770	0.990
VEGF	0.021	0.009	5.359	0.021	1.021	1.003	1.039

### ROC survival curve analysis revealed the diagnostic efficacy of cytokines for breast malignancies

**Figure 1:**
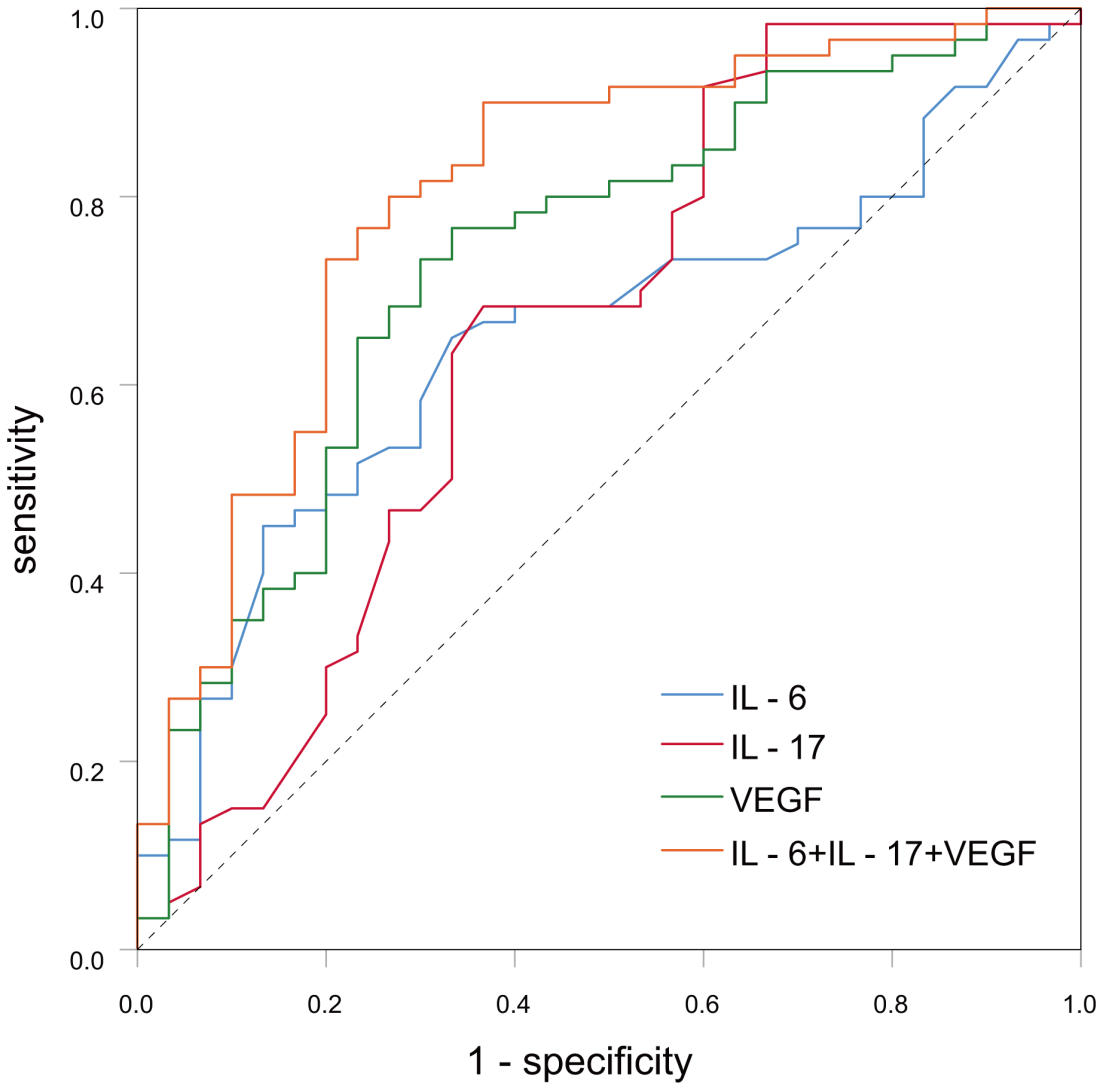
The curve graph of serum IL-6, IL-17, VEGF in breast cancer and benign breast disease patient.

ROC curve analysis showed that IL-6, IL-17 and VEGF formed an AUC on the ROC curve of 0.649 (95% CI: 0.534-0.765, p-value < 0.05), 0.656 (95% CI: 0.517-0.786) and 0.734 (95% CI: 0.622-0.845, p-value < 0.001). Sensitivity was 65%, 91.7%, and 76.7%, and specificity was 66.7%, 40%, and 66.7%. Yoden index was. 0.317, 0.317, and 0.434. (**Table 3**; **Figure 1**). However, the AUC was 0.801 (95% CI: 0.699-0.903, p-value < 0.001), sensitivity was 83.3%, and specificity was 73.3% when IL-6, IL-17, and VEGF were combined for diagnosis, therefore, serum levels of IL-6, IL-17 and VEGF are associated with the development and progression of breast cancer, and combined monitoring of the above cytokines seems to have clinical application for the early diagnosis of breast cancer patients. (**Table 3**; **Figure 1**).

**Table 3:** ROC analysis of serum IL-6, IL-17 and VEGF in differentiating breast cancer from benign control group.

Indicators	AUC	Sensitivity	Specificity	Yoden Index	Threshold	P-value
IL-6	0.649 (0.534-0.765)	65%	66.70%	0.317	0.73	**0.021**
VEGF	0.734 (0.622-0.845)	76.70%	66.70%	0.434	20.09	**<0.001**
IL-17	0.656 (0.517-0.786)	91.7%	40%	0.317	0.735	**0.016**
Joint Diagnosis	0.801 (0.699-0.903)	83.3%	73.3%	0.566	0.527	**<0.001**

AUC, Area under the curve. Joint Diagnosis: Co-diagnosis with IL-6, IL17 and VEGF.

Values in bold represent statistically significant results (P<0.05).

### Relationship between serum cytokine levels of breast cancer patients and clinical characteristics of breast cancer patients

There was no statistically significant difference between serum cytokine levels and lymph node metastasis in breast cancer patients (P>0.05). Analyzed from the perspective of clinical stage of breast cancer, there was no significant correlation between serum cytokine levels and TNM stage in breast cancer patients. We also further distinguished whether there was any difference in serum cytokine levels between different types of breast cancer in different lymph node metastasis situations and different TNM stages, and the results of the study showed no statistical difference either (P>0.05). (**Tables 4**, **5**).

**Table 4:** Serum IL-5, IL-6, IL-8, IL-17, VEGF levels and lymph node metastasis in breast cancer patients.

Indicators	Presence of lymph node metastasis		P-value
	No lymph node metastasis group	Group with lymph node metastasis	
IL-5	0.97 (0.38-2.03)	1.40 (0.43-2.53)	0.432
IL-6	1.03 (0.40-2.50)	1.30 (0.47-2.31)	0.980
IL-8	1.64 (0.81-4.73)	0.95 (0.24-19.12)	0.713
IL-17	0.39 (0.18-0.88)	0.47 (0.18-0.97)	0.947
VEGF	37.70 (23.00-75.80)	33.12 (13.57 - 88.81)	0.367

**Table 5:** Cytokines associated with TNM staging.

Indicators	TNM Staging		P-value
	Phase I	Phase II - IV	
IL-5	0.92 (0.33-2.04)	1.12 (0.43-2.47)	0.624
IL-6	1.12 (0.54-2.11)	0.74 (0.36-2.88)	0.716
IL-8	1.79 (0.90-5.69)	0.97 (0.37-3.27)	0.181
IL-17	0.39 (0.17-0.79)	0.45 (0.19-0.99)	0.806
VEGF	36.70 (21.02-105.63)	35.64 (18.50-70.84)	0.727

### Relationship between serum cytokine expression levels and molecular typing of breast cancer patients

In this study, a total of 60 patients in the breast cancer group were divided into three groups according to the molecular typing method, including 25 patients in the hormone receptor-positive group, 25 patients in the HER-2-positive group, and 10 patients in the triple-negative group, in combination with intraoperative or postoperative immunohistochemistry of the patients. The KW test revealed that there was no significant difference in cytokine expression levels between the IL-5, IL-6 and IL-8 groups (P values of 0.396, 0.050 and 0.551, respectively), while there was a significant difference in cytokine expression levels between the IL-17 and VEGF groups (P values of 0.008 and 0.040, respectively). The expression levels of IL-17 and VEGF were higher in HER-2 receptor-positive and triple-negative patients than in hormone receptor-positive patients (P < 0.05), while there was no significant difference in the expression levels of the above cytokines between triple-negative and HER-2 receptor-positive patients. (**Table 6**).

**Table 6:** Comparison of serum IL-5, IL-6, IL-8, IL-17 and VEGF levels in the breast cancer surgery group before and after surgery.

Indicators	Before Surgery	After Surgery	P-value
IL-5	1.01 (0.38-2.19)	1.02 (0.35-2.23)	0.840
IL-6	1.11 (0.45-2.44)	2.87 (1.16-8.22)	**<0.001**
IL-8	1.47 (0.79-4.73)	1.07 (0.44-2.98)	0.413
IL-17	0.40 (0.18-0.88)	0.55 (0.24-0.93)	0.200
VEGF	35.78 (20.73-75.80)	25.63 (16.74-43.63)	**0.045**

Values in bold represent statistically significant results (P<0.05).

### Changes in serum cytokine expression levels in breast cancer patients before and after surgery

In the breast cancer surgery group, postoperative VEGF levels were statistically significantly lower compared to preoperative levels (P < 0.05); postoperative IL-6 levels were statistically significantly higher compared to preoperative levels (P < 0.05). In contrast, the expression levels of IL-5, IL-8 and IL-17 before and after surgery were not statistically significant (P > 0.05) (**Table 7**).

**Table 7:** Cytokines associated with molecular typing.

Cytokines	Hormone receptor-positive type (n=25)	Her-2-positive type(n=25)	Triple negative type(n=10)	P-value	P1	P2	P3
IL-5	0.92 (0.33-2.28)	1.07 (0.33-1.89)	1.30 (0.81-3.36)	0.396	–	–	–
IL-6	1.66 (0.64-2.49)	0.65 (0.25-2.10)	1.82 (0.74-4.42)	0.050	–	–	–
IL-8	1.61 (0.39-3.27)	1.47 (0.80-9.34)	0.99 (0.66-3.03)	0.551	–	–	–
IL-17	0.27 (0.14-0.50)	0.46 (0.22-1.06)	0.72 (0.44-1.15)	0.008	0.038	0.169	0.003
VEGF	26.12 (15.97-41.42)	41.20 (23.21 - 113.88)	44.08 (34.47 - 142.11)	0.040	0.046	0.485	0.028

P1, Difference in cytokines between hormone receptor-positive and Her-2 receptor-positive types. P2, Difference in cytokines between Her-2 receptor-positive and triple-negative types. P3, Difference in cytokines between hormone receptor-positive and triple-negative types. p<0.05 was considered statistically significant.

## Discussion

Breast cancer, as one of the most common female malignancies, has insidious early symptoms and lacks effective, sensitive, and specific early diagnosis methods. The relationship between the tumor and inflammation has attracted more attention. In addition to cancer cells, 80% of the cells in tumor tissue belong to mesenchymal and inflammatory cells. These mesenchymal cells produce cytokines that form a complex network involved in the regulation and development of tumors (7). We analyzed the serum samples of the enrolled patients and found that some changes in the levels of cytokines in breast cancer patients may have interesting implications.

IL-6 is significantly highly expressed in the stroma of some malignancies, such as in prostate and colorectal cancers where it can influence tumor growth and differentiation (8–11). In breast tumors, Iliopoulos et al. (12) showed that Src, an oncogene of the non-receptor tyrosine kinase family, induces the transformation of normal mammary epithelial cells through activation of NF-κB, and IL-6 plays an important regulatory role in this process.There are also studies indicating that IL-6 is associated with TNM staging, recurrence and metastasis of breast cancer (13). The results of this paper showed that IL-6 expression levels were significantly higher in the breast cancer group compared to the benign breast tumor group, and serum IL-6 expression levels were significantly higher in patients with HER-2 (+) and triple negative breast cancer than in patients with HR (+). A significant increase in IL-6 expression in patients with HER-2(+) and triple-negative breast cancer may indicate a poor prognosis. The mechanism of its elevation may be that the patients themselves may have cellular immune dysfunction, resulting in a dysregulated lymphocyte ratio, or that the inflammatory response is exacerbated by macrophage phagocytosis or cytosolic drinking of antigenic substances in the patient’s body, prompting monocytes to produce large amounts of IL-6. Whether there are other factors influencing this remains to be further explored. The high expression of IL-6 in the present study seems to suggest a close association with tumor aggressiveness, and blocking the cancer-associated inflammatory factor IL-6 alone or in combination with conventional anticancer therapies to inhibit its associated signaling may be a potential therapeutic strategy for the treatment of cancers in which IL-6 is the dominant signal. Also, its expression level may be of value in monitoring the status of breast cancer patients.

In recent years, IL-17 cells have been identified in a variety of tumors, including breast cancer (6, 14). The number of IL-17 cells is significantly increased in tumors compared to normal segments of tumor tissue in patients (15, 16). It has been reported that IL-17 may play a dual role in tumor progression due to the complex mechanism of interaction with tumors (17–19). Chen et al,found that high expression of IL-17 in tumors significantly improved the 5-year overall survival of patients with gastric adenocarcinoma (20). Meanwhile, Liu et al, in a study on colorectal cancer found that IL-17 overexpression may be involved in active tumor microangiogenesis through its signaling pathway, promoting tumor growth (21). In our study, reduced IL-17 expression was found to be an independent risk factor for patients with malignant breast tumors. And when different molecular typing was compared with each other, serum IL-17 expression levels were found to be significantly higher in patients with HER-2 breast cancer and triple negative breast cancer than in HR-positive type. The above results also favor the inference that IL-17 has a dual role in tumors, with significantly higher IL-17 expression levels in patients with benign breast tumors than in malignant breast tumors; however, in patients with malignant breast tumors, high IL-17 expression may increase tumor aggressiveness by participating in signaling pathways such as tumor microangiogenesis, which is similar to the findings of Liu et al. (21). In addition, studies by Benevides et al. (22) and Laprevott et al. (23) also showed that high IL-17 expression promotes breast cancer progression and is associated with a poorer prognosis of breast cancer.

VEGF has been found to be highly expressed in various tumors and body fluids, such as breast and rectum. Currently, VEGF is considered to be the most important regulator of the process known to promote angiogenesis (3). Reports on the role of VEGF in breast cancer are inconclusive. It has been suggested that high levels of VEGF may influence the biomorphology of tumors and it may promote lymph node metastasis or distant metastasis (24). Also, a group of authors showed a correlation between high VEGF expression and shorter survival of patients (25, 26). Some other authors noted a correlation between high levels of VEGF, tumor size and regional lymph node metastasis. Our data found no significant correlation between VEGF concentration and patients’ primary tumor size, lymph node metastasis, and tumor TNM stage. And VEGF expression level is significantly different only in benign and malignant breast tumors. Also in patients with different molecular staging of breast cancer, VEGF expression levels were found to be significantly higher in the triple-negative and HER-2 receptor-positive types than in the hormone receptor-positive type. The present study showed that there seems to be a correlation between hormone receptor deficiency and HER-2 overexpression and serum VEGF expression levels, which is consistent with the literature findings such as the association of high VEGF expression with poor patient prognosis (26). The above results suggest that VEGF is closely related to the development and invasive metastasis of breast cancer and can be used as a monitoring indicator for clinical prognosis and efficacy assessment. In addition, in the treatment of breast cancer, to inhibit the growth of cancer cells, it can be achieved by blocking VEGF-mediated endothelial cell mitosis and reducing the formation of tumor neovascularization. Therefore, the monitoring of serum VEGF in breast cancer patients is important, not only for the assessment of disease progression, but also as an effective target for clinical treatment.

To our knowledge, there is little high-level evidence for a role of IL-5 in cancer. Quail DF et al. (27) showed that obese patients increased the likelihood of lung metastasis from breast cancer by upregulating serum IL-5 expression levels by exacerbating the metastatic process from neutrophils to the lung (28, 29). Also in bladder cancer, elevated IL-5 levels enhance bladder cancer cell migration and invasion through the extracellular signal-regulated kinase 1/2-mediated matrix metallopeptidase 9/nuclear factor κ/activator 1 pathway (30). However, our study found no significant correlation between IL-5 expression levels and the pathological characteristics of breast cancer patients. Meanwhile, IL-8, a CXC family pro-inflammatory cytokine responsible for recruitment and chemotactic response at the site of inflammation, can also be autocrine by tumour cells and promote tumour progression.Waugh DJ et al. showed that in non-hormone-dependent breast cancer, IL-8 can lead to enhanced migration of breast cancer through activation of the PI3K/Akt pathway as well as the PLC/PKC pathway (31, 32).K önig A et al. (4) showed that in cases with tumour grade G3 and progesterone receptor negativity, elevated IL-8 can promote tumour metastatic spread by promoting neovascularisation (33, 34). However, in our collective study, there was also no significant correlation regarding IL-8 with the pathological characteristics of breast cancer patients. This finding may be related to the fact that this paper mainly studied patients with stage I and II early-stage breast cancer, most of whom had a good prognosis as treatment progressed, but the lack of a group of patients with poorer prognosis in stages III and IV in this study may have influenced the final conclusions. Given the importance of Th2 cytokines in promoting cancer progression in the primary tumour microenvironment, studies need to be refined to further understand the link between the aforementioned factors and breast cancer.

Our study has some potential limitations. First, this was a retrospective single-center study with a small sample size, especially for patients with advanced breast cancer. A study with a large population is necessary to confirm the present results. Second, in our study, we only collected two venous blood samples from every patients to send for examination, and patients should be followed up for a long time for the above cytokine changes and patients’ long-term survival status to explore the correlation in depth. These works can provide the basis for further diagnostic studies.

## Conclusion

The results of this paper showed that among breast cancers of different molecular stages, IL-6, IL-17 and VEGF were significantly higher in the breast cancer group than in the group with benign breast disease, and IL-17 and VEGF were higher in the HER-2 receptor-expressing and triple-negative types than in the hormone receptor-positive group. However, the present study showed no significant correlation between the expression levels of the above cytokines and the stage of disease and axillary lymphatic metastasis. The experimental analysis of this study showed that serum IL-6, IL-17 and VEGF levels are closely associated with the development, progression and invasion of breast cancer and can be used as monitoring indicators for clinical prognosis and efficacy assessment. In contrast, IL-5 and IL-8 have not been found to be significantly associated with breast cancer in this study, and we can further explore his assessment efficacy by expanding the sample size subsequently. In conclusion, the association between serum cytokines and tumor immunity and disease characteristics of breast cancer patients needs further study, and the search for early diagnostic indicators and postoperative follow-up indicators with better efficacy is an important of future research.

## Data availability statement

The raw data supporting the conclusions of this article will be made available by the authors, without undue reservation.

## Ethics statement

The studies involving human participants were reviewed and approved by the institutional ethics committee of the First Affiliated Hospital of Chongqing Medical University. Written informed consent for participation was not required for this study in accordance with the national legislation and the institutional requirements.

## Author contributions

All authors contributed to data analysis, drafting, and revising the article; gave final approval of the version to be published; and agreed to be accountable for all aspects of the work. All authors contributed to the article and approved the submitted version.

## Funding

This study was supported by the Research Fund of the First Affiliated Hospital of Chongqing Medical University.

## Acknowledgments

The authors thank all the patients who participated in this study.

## Conflict of interest

The authors declare that the research was conducted in the absence of any commercial or financial relationships that could be construed as a potential conflict of interest.

## Publisher’s note

All claims expressed in this article are solely those of the authors and do not necessarily represent those of their affiliated organizations, or those of the publisher, the editors and the reviewers. Any product that may be evaluated in this article, or claim that may be made by its manufacturer, is not guaranteed or endorsed by the publisher.
