# Supplementary material for: Expression analysis of cytokines IL-5, IL-6, IL-8, IL-17 and VEGF in breast cancer patients
Source: Front Oncol. 2022 Dec 1;12:1019247. doi: 10.3389/fonc.2022.1019247 (PMC9753904; doi:10.3389/fonc.2022.1019247)
Supplement: Supplementary file 1 [file Table_1.docx]

| TABLE NAME | STATISCAL MODELS |
| --- | --- |
| TABLE 1 | Single factor analysis |
| TABLE 2 | Multi-factor logistic regression analysis |
| TABLE 3 | ROC curve analysis |
| TABLE 4 | Wilcoxon (Mann-Whitney U) test |
| TABLE 5 | Wilcoxon (Mann-Whitney U) test |
| TABLE 6 | Wilcoxon matched-pairs signed rank test |
| TABLE 7 | Wilcoxon (Kruskal-Wallis) test |

1.A supplementary table:with description of statistical models for each compared phenotype
